# Semantic Predictability Facilitates Comprehension of Degraded Speech in a Graded Manner

**DOI:** 10.3389/fpsyg.2021.714485

**Published:** 2021-09-09

**Authors:** Pratik Bhandari, Vera Demberg, Jutta Kray

**Affiliations:** ^1^Department of Psychology, Saarland University, Saarbrücken, Germany; ^2^Department of Language Science and Technology, Saarland University, Saarbrücken, Germany; ^3^Department of Computer Science, Saarland University, Saarbrücken, Germany

**Keywords:** speech perception, language comprehension, bottom-up processing, top-down prediction, semantic prediction, probabilistic prediction, perceptual adaptation, noise-vocoded speech

## Abstract

Previous studies have shown that at moderate levels of spectral degradation, semantic predictability facilitates language comprehension. It is argued that when speech is degraded, listeners have *narrowed expectations* about the sentence endings; i.e., semantic prediction may be limited to only most highly predictable sentence completions. The main objectives of this study were to (i) examine whether listeners form narrowed expectations or whether they form predictions across a wide range of probable sentence endings, (ii) assess whether the facilitatory effect of semantic predictability is modulated by perceptual adaptation to degraded speech, and (iii) use and establish a sensitive metric for the measurement of language comprehension. For this, we created 360 German Subject-Verb-Object sentences that varied in semantic predictability of a sentence-final target word in a graded manner (high, medium, and low) and levels of spectral degradation (1, 4, 6, and 8 channels noise-vocoding). These sentences were presented auditorily to two groups: One group (*n* =48) performed a listening task in an unpredictable channel context in which the degraded speech levels were randomized, while the other group (*n* =50) performed the task in a predictable channel context in which the degraded speech levels were blocked. The results showed that at 4 channels noise-vocoding, response accuracy was higher in high-predictability sentences than in the medium-predictability sentences, which in turn was higher than in the low-predictability sentences. This suggests that, in contrast to the *narrowed expectations* view, comprehension of moderately degraded speech, ranging from low- to high- including medium-predictability sentences, is facilitated in a graded manner; listeners probabilistically preactivate upcoming words from a wide range of semantic space, not limiting only to highly probable sentence endings. Additionally, in both channel contexts, we did not observe learning effects; i.e., response accuracy did not increase over the course of experiment, and response accuracy was higher in the predictable than in the unpredictable channel context. We speculate from these observations that when there is no trial-by-trial variation of the levels of speech degradation, listeners adapt to speech quality at a long timescale; however, when there is a trial-by-trial variation of the high-level semantic feature (e.g., sentence predictability), listeners do not adapt to low-level perceptual property (e.g., speech quality) at a short timescale.

## Introduction

Understanding speech is highly automatized and seemingly easy when conditions are optimal. However, in our day-to-day communication, conditions are often far from being optimal. Intelligibility and comprehension of speech can be compromised at the source (speaker), at the receiver (listener), and at the transmission of the speech signal (environmental factor; Shannon, 1948). Ambient noise is an environmental factor that distorts the speech signal and renders it difficult to understand. For example, the noise coming from people talking in the background might make it difficult for you to understand what your friend is saying, while you are chatting in a café. Similarly, a conversation with a friend over the phone can be corrupted by a poor transmission of the speech signal which in turn hampers language comprehension. Interestingly, although the speech signal is sometimes bad or the environment is noisy, listeners do not always fail to understand what a friend is saying in the café or over the phone. Instead, listeners are successful in understanding distorted speech by utilizing context information which contains information in a given situation about a topic of conversation, semantic and syntactic information of a sentence structure, world knowledge, visual information, etc. (Kaiser and Trueswell, 2004; Knoeferle et al., 2005; Altmann and Kamide, 2007; Xiang and Kuperberg, 2015; for reviews, Stilp, 2020). The goals of the present study were threefold: First, to examine the interplay between perceptual and cognitive processing during language comprehension to answer the question whether the predictability of the sentence context facilitates language comprehension in a graded manner primarily when the speech signal is distorted, second, to assess whether perceptual adaptation influences the interplay between perceptual and language processing, and third, to establish a sensitive metric that takes into account the use of context in language comprehension.

### Language Comprehension and Sentence Context (Predictability)

Research from various domains of cognitive (neuro)science, like emotion, vision, odor, and proprioception, has shown that predicting upcoming events influences human perception and cognition (Stadler et al., 2012; Clark, 2013; Seth, 2013; Marques et al., 2018). There is also a large body of evidence from psycholinguistics and cognitive neuroscience of language, suggesting that human language comprehension is predictive in nature (Lupyan and Clark, 2015; Pickering and Gambi, 2018; see also Huettig and Mani, 2016). Empirical evidence from a number of studies suggests that readers or listeners predict upcoming words in a sentence when the words are predictable from the preceding context (for reviews, Staub, 2015; Kuperberg and Jaeger, 2016). For instance, words that are highly predictable from the preceding context are read faster and are skipped compared to the less predictable ones (Ehrlich and Rayner, 1981; Frisson et al., 2005; Staub, 2011). Applying the visual world paradigm, several studies have demonstrated that participants show anticipatory eye movements toward the picture of the word predictable from the sentence context (Altmann and Kamide, 1999; Kamide et al., 2003; Jachmann et al., 2019). The sentence-final word in a highly predictable sentence context (e.g., “*She dribbles a ball*.”) elicits a smaller N400, a negative going EEG component that peaks around 400ms post-stimulus and is considered as a neural marker of context-based expectation, than that in a less predictable sentence context (e.g., “*She buys a ball*.”; Kutas and Hillyard, 1984; Federmeier et al., 2007; for reviews, see Kutas and Federmeier, 2011; see also Brouwer and Crocker, 2017). Similarly, event-related words (e.g., “luggage”) elicited reduced N400 compared to event-unrelated words (e.g., “vegetables”) which were not predictable from the context (e.g., in a “travel” scenario; Metusalem et al., 2012). In sum, as the sentence context builds up, listeners make predictions about upcoming words in the sentence, and these in turn facilitate language comprehension. Here, we will investigate whether individuals make use of the predictability of the sentence context when perceptual processing is hampered due to a bad quality of the speech signal.

### Language Comprehension Under Reduced Quality of the Speech Signal

The detrimental effect of distortion of speech signal in language comprehension and speech intelligibility has been investigated for several types of artificial distortions, like multi-talker babble noise, reverberation, time compression, and noise-vocoding. For instance, it has been shown that speech intelligibility and comprehension decreases (a) with a decrease in signal-to-noise ratio under multi-talker babble noise conditions (e.g., Fontan et al., 2015), (b) faster rate of speech (e.g., Wingfield et al., 2006), and (c) longer reverberation time (e.g., Xia et al., 2018).

Similarly, noise-vocoding also impedes speech intelligibility. Noise-vocoding is an effective method to parametrically vary and control the intelligibility of speech in a graded manner. Noise-vocoding distorts speech by dividing a speech signal into specific frequency bands corresponding to the number of vocoder channels. The frequency bands are analogous to the electrodes of cochlear implant (Shannon et al., 1995, 1998). The amplitude envelope within each band is extracted and is used to modulate noise of the same bandwidth. This renders vocoded speech harder to understand by replacing the fine structure of the speech signal with noise while preserving the temporal characteristics and periodicity of perceptual cues. With the increase in number of channels, more frequency-specific information becomes available, spectral resolution of the speech signal increases, and hence, speech becomes more intelligible; for example, speech processed through 8 channels noise-vocoding is more intelligible than the one processed through 4 channels noise-vocoding (Loizou et al., 1999; Shannon et al., 2004). The level of degradation, i.e., the number of channels used for noise-vocoding, required for the same level of task accuracy can vary from 3 to 30 or more depending on the method implemented for noise-vocoding (e.g., Ueda et al., 2018), participant variables (age and language experience), test materials (words, sentences, and accented speech), and listening conditions (speech in quiet and speech with background noise; Shannon et al., 2004). Here, we will systematically vary the level of speech degradation by noise-vocoding of unaccented speech to determine whether listeners benefit from the sentence context for language comprehension when the signal quality is not too bad or too good, hence at moderate levels of speech degradation.

### Predictive Processing and Language Comprehension Under Degraded Speech

Top-down predictive and bottom-up perceptual processes interact dynamically in language comprehension. In a noisy environment, when the bottom-up perceptual input is less reliable, it has been shown that participants rely more on top-down predictions by narrowing down the predictions to smaller sets of semantic categories or words (e.g., Strauß et al., 2013; see also Corps and Rabagliati, 2020). Obleser and colleagues (Obleser et al., 2007; Obleser and Kotz, 2010, 2011), for instance, used sentences of two levels of semantic predictability (high and low) and systematically degraded the speech signal by passing it through various numbers of noise-vocoding channels ranging from 1 to 32 in a series of behavioral and neuroimaging studies. They found that semantic predictability facilitated language comprehension at a moderate level of speech degradation. That is, participants relied more on the sentence context when the speech signal was degraded but intelligible enough than when it was not degraded or was highly degraded. At such moderate levels of speech degradation, accuracy of word recognition was found to be higher for highly predictable target words than for less expected target words (Obleser and Kotz, 2010). For the extremes, i.e., when the speech signal was highly degraded or when it was clearly intelligible, the word recognition accuracy was similar across both levels of sentence predictability, meaning that predictability did not facilitate language comprehension. The conclusion of these findings is that at moderate levels of degradation, participants rely more on the top-down prediction generated by the sentence context and less on the bottom-up processing of unclear, less reliable, and degraded speech signal (Obleser, 2014). Reliance on prediction results in higher word recognition accuracy for target words with high-cloze probability than for the target words with low-cloze probability. In the case of a heavily degraded speech signal, participants may not be able to understand the sentence context and, therefore, be unable to form predictions of the target word, or their cognitive resources may already be occupied by decoding the signal, leaving little room for making predictions. Thus, there is no differential effect of levels of sentence predictability. On the other extreme, when the speech is clear and intelligible (at the behavioral level, i.e., when the participants respond what the target word of the sentence is), participants recognize the intelligible target word across all levels of sentence predictability. Hence, no differential effect of levels of predictability of target word can be expected.

These findings of Obleser and colleagues (Obleser and Kotz, 2011) were replicated and extended by Strauß et al. (2013; see Obleser, 2014). In a modified experimental design, they varied the target word predictability by manipulating its expectancy (i.e., how expected the target word is given the verb) and typicality (i.e., co-occurrence of target word and the preceding verb). They reported that at a moderate level of spectral degradation, N400 responses at strong-context, low-typical words and weak-context, low-typical words were largest. N400 responses at the latter two were not statistically different from each other. However, the N400 response was smallest at highly predictable (strong-context and high-typical) words. The authors interpreted these findings as a facilitatory effect of sentence predictability which might be limited to only highly predictable sentence endings at a moderate level of spectral degradation. In their “expectancy searchlight model,” they suggested that listeners form “narrowed expectations” from a restricted semantic space when the sentence endings are highly predictable. When the sentence endings are less predictable, listeners cannot preactivate those less predictable sentence endings in an adverse listening condition. This is contrary to the view that readers and listeners form a probabilistic prediction of upcoming word in a sentence. For example, Nieuwland et al. (2018) showed in a large-scale replication study of DeLong et al. (2005) that the N400 amplitude at the sentence-final noun is directly proportional to its cloze probability across a range of high- and low-cloze words (see also, Kochari and Flecken, 2019; Nicenboim et al., 2020). Heilbron et al. (2020) also showed that a probabilistic prediction model outperforms a constrained guessing model, suggesting that linguistic prediction is not limited to highly predictable sentence endings, but it operates broadly in a wide range of probable sentence endings. However, a difference is that these studies were conducted in conditions without noise.

The probabilistic nature of prediction in comprehension of degraded speech has focused on a comparison of listeners’ response to high-cloze target words and low-cloze target words (Obleser et al., 2007; Obleser and Kotz, 2011; see also, Strauß et al., 2013; Amichetti et al., 2018). In the present study, we included sentences with medium-cloze target words (see Supplementary Material). If the listeners form a narrowed prediction only for high-cloze target words, then the facilitatory effect of semantic prediction will be observed only at these highly predictable sentence endings. Listeners’ response to medium-cloze target words and low-cloze target words would be expected to be quite similar as these two will fall out of the range of narrowed prediction. However, if the listeners’ predictions are not restricted to highly predictable target words, then they form predictions across a wide range of semantic context proportional to the probability of occurrence of the target word. In addition to highly predictable sentence endings, listeners will also form predictions for less predictable sentence endings. Such predictions, however, will depend on the probability of occurrence of the target words. In other words, listeners form predictions also for less expected sentence endings; and the semantic space of prediction depends on the probability of occurrence of those sentence endings. The addition of sentences with medium-cloze target words in the present study thus allows us to differentiate whether listeners form all-or-none prediction restricted to high-cloze target words, or a probabilistic prediction for words across a wide range of cloze probability.

### Adaptation to Degraded Speech

Listeners quickly adapt to novel speech with artificial acoustic distortions (e.g., Dupoux and Green, 1997). Repeated exposure to degraded speech leads to improved comprehension over time (for reviews, Samuel and Kraljic, 2009; Guediche et al., 2014). When the noise condition is constant throughout the experiment, listeners adapt to it and the performance (e.g., word recognition) improves with as little as 20min of exposure (Rosen et al., 1999). For example, Davis et al. (2005, Experiment 1) presented listeners with sentences with 6 channels of noise-vocoding and found an increase in the proportion of correctly reported words over the course of experiment. Similarly, Erb et al. (2013) presented participants with sentences passed through 4 channels of noise-vocoding and reached a similar conclusion. In these experiments, only a single spectrally degraded speech signal (passed through 6 or 4 channels) was presented in one block. Therefore, it was predictable from the point of view of the participant which level of spectral degradation will appear in any trial within the block. Additionally, target word predictability was not varied.

When multiple types or levels of degraded speech signals are presented in a (pseudo-)randomized order within a block, then a listener is uncertain about any upcoming trials’ signal quality; if such multiple levels of degradation are due to the presentation of multiple channels of noise-vocoded speech, then the global channel context is unpredictable or uncertain. This can influence perceptual adaptation. For instance, Mattys et al. (2012) note the possibility for a total absence of perceptual adaptation, when the characteristics of auditory signal change throughout an experiment. We also know from Sommers et al. (1994) that trial-by-trial variability in the characteristics of distorted speech impairs word recognition (see also, Dahan and Magnuson, 2006). We thus speculated that if the noise-vocoded speech varies from one trial to the next, then the adaptation to noise in this scenario might be different from the earlier case in which spectral degradation is constant throughout the experiment. Perceptual adaptation, however, is not limited to trial-by-trial variability of stimulus property. Listeners can adapt to auditory signal at different time courses and time scales (Atienza et al., 2002; see also, Whitmire and Stanley, 2016). In addition to differences in intrinsic trial-by-trial variability and resulting short timescale trial-by-trial adaptation in two channel contexts, the global differences in the presentation of vocoded speech can result in a difference in the general adaptation at a longer timescale between predictable and unpredictable channel contexts.

There is a limited number of studies that has looked at how next-trial noise-uncertainty and global context of speech property influence adaptation. For example, words were presented at +3dB SNR and +10dB SNR in a word-recognition task in a pseudorandom order (Vaden et al., 2013). The authors wanted to minimize the certainty about the noise conditions in the block. The same group of authors (Vaden et al., 2015, 2016; Eckert et al., 2016) proposed that an adaptive control system (cingulo-opercular circuit) might be involved to optimize task performance when listeners are uncertain about the upcoming trial. However, we cannot make a firm conclusion about perceptual adaptation *per se* from their studies as they do not report the change in performance over the course of experiment. Similarly, Obleser and colleagues (Obleser et al., 2007; Obleser and Kotz, 2011; Hartwigsen et al., 2015) also presented listeners with noise-vocoded sentences (ranging from 2 to 32 channels of noise-vocoding) in a pseudo-randomized order but did not report the presence or absence of perceptual adaptation to noise-vocoded speech. In the above-mentioned studies, the authors did not compare participants’ task performance in blocked design against the presentation in a pseudorandomized block of different noise conditions to make an inference about general adaptation to degraded speech at a longer timescale. To examine the influence of uncertainty about next-trial speech features and the global context of speech features on perceptual adaptation, we will therefore compare language comprehension with a trial-by-trial variation of sentence predictability and speech degradation either in blocks in which the noise-vocoded channels are blocked, or in a randomized order.

### Measurement of Language Comprehension

Another issue we would like to discuss is how to best measure language comprehension. The measurement of comprehension performance is inconsistent across studies. For instance, Erb et al. (2013) and Hakonen et al. (2017) measured participants’ performance as proportion of correctly reported words per sentence (“report scores”; Peelle et al., 2013). On the other hand, Sheldon et al. (2008) asked participants to only report the final word of the sentence and then calculated the proportion of correctly reported words. One disadvantage of these approaches is that they do not consider whether participants correctly identified the sentence context or not. A crude word recognition score and the proportion of correct responses do not reflect an accurate picture of facilitation (or lack thereof) of language comprehension. Therefore, in the present study, we consider only those responses in which participants correctly identify the sentence context.

### Goals of This Study

In sum, the goals of the study were threefold. Our first goal was to replicate and extend the behavioral results of Obleser and colleagues (Obleser et al., 2007; Obleser and Kotz, 2011), namely that the effect of semantic predictability will be observed only at a moderate level of speech degradation – as participants can realize the sentence context at the moderate level, their prediction will be narrowed down and the reliance on the bottom-up processing of the sentence final word will be over-ridden by top-down prediction. In contrast, when the sentence is clearly intelligible, even if the prediction is narrowed down and regardless of whether the clearly intelligible final word confirms or disconfirms those predictions, they respond based on what they hear, i.e., they rely mostly on acoustic-phonetic rather than lexical cues. Our study will provide new insights to the field by examining whether listeners indeed form *narrowed expectations* such that the facilitatory effect of predictability will be observed only for high-cloze target words and not at medium- or low-cloze probability. To examine this, we created 360 German sentences at different levels of target word probability – low-cloze probability=0.022±0.027, medium-cloze probability=0.1±0.55, and high-cloze probability=0.56±1.0 – and varied the levels of spectral degradation by noise-vocoding through 1, 4, 6 and 8 channels.

Our second goal was to investigate the role of an uncertainty about the next-trial speech features on perceptual adaptation by varying the global channel context on the comprehension of degraded speech. To study this, we presented sentences of different levels of predictability blocked by each channel conditions (*predictable channel context*) and pseudo-randomized across all channels (*unpredictable channel context*). Based on previous findings, we expected that in the unpredictable channel context (i.e., when sentences are presented in a random order of spectral degradation) participants’ word recognition performance will be worse than in the predictable channel context (i.e., when the sentences are blocked by noise-vocoding; Sommers et al., 1994; Garrido et al., 2011; Vaden et al., 2013). Moreover, to further examine perceptual adaptation, we also considered the effect of trial number in the analyses of data.

Our third goal was to establish a sensitive metric of measurement of language comprehension which considers the use of context by listeners. We note the caveat in the measurement of language comprehension in above-mentioned studies (e.g., Sheldon et al., 2008; Erb et al., 2013; Hakonen et al., 2017) and extend it further with a metric that we consider is a better measure of language comprehension (Amichetti et al., 2018) in the write-down paradigm (Samar and Metz, 1988).

## Materials and Methods

### Participants

We recruited two groups of participants *via* Prolific Academic and assigned them to one of the two groups: “unpredictable channel context” (*n* =48; x̅±SD=24.44±3.5years; age range=18–31years; 16 females) and “predictable channel context” (*n* =50; x̅±SD=23.66±3.2years; age range=18–30years; 14 females). All participants were native speakers of German residing in Germany. Exclusion criteria for participating in this study were self-reported hearing disorder, speech-language disorder, or any neurological disorder. All participants received monetary compensation for their participation. The study was approved by the Deutsche Gesellschaft für Sprachwissenschaft (DGfS) Ethics Committee, and the participants provided consent in accordance with the Declaration of Helsinki.

### Stimuli

**Figure 1:**
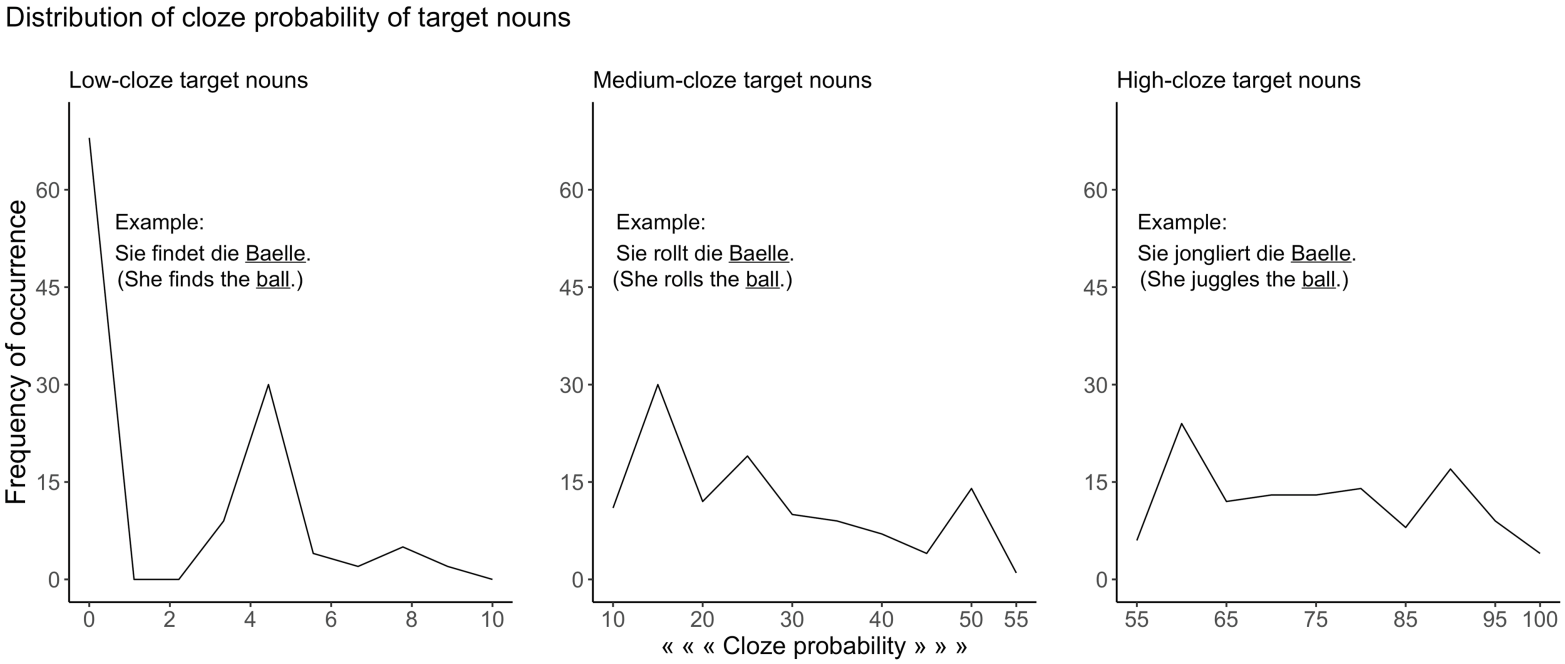
The distribution of cloze probability values for low-, medium-, and high-cloze target nouns. Example sentences with their English translations are shown on each plot.

The stimuli were digital recordings of 360 German sentences spoken by a female native speaker of German in a normal rate of speech. All sentences were in present tense consisting of pronoun, verb, determiner, and object (noun) in the Subject-Verb-Object form. We used 120 unique nouns to create three sentences that differed in cloze probability of target words. This resulted into sentences with low-, medium-, and high-cloze target word (for examples, see Figure 1).

We collected cloze probability ratings for each of these sentences in separate groups of younger participants (*n* =60) of the same age range (18–30years) prior to this study, while not all participants received the full set of 360 sentences. Mean cloze probabilities were 0.022 (SD=0.027; range=0.00–0.09) for sentences with low-cloze target word (low-predictability sentences), 0.274 (SD=0.134; range=0.1–0.55) for sentences with medium-cloze target word (medium-predictability sentences), and 0.752 (SD=0.123; range=0.56–1.00) for sentences with high-cloze target word (high-predictability sentences). The distribution of cloze probability across low-, medium-, and high-predictability sentences is shown in Figure 1.

The sentences were recorded and digitized at 44.1kHz with 32-bit linear encoding. The spectral degradation conditions of 1, 4, 6, and 8 channels were achieved for each of the 360 recorded sentences using a customized noise-vocoding script originally written by Darwin (2005) in Praat. The speech signal was divided into 1, 4, 6, and 8 frequency bands between 70 and 9,000Hz. The frequency boundaries were determined by cochlear-frequency position functions, and the boundary frequencies were approximately logarithmically spaced (Greenwood, 1990; Erb, 2014). The amplitude envelope of each band was extracted and applied to band-pass filtered white noise in the same frequency ranges; the upper and lower bounds for band extraction are specified in Table 1. Each of the modulated noises was then combined to produce distorted sentence. Scaling was performed to equate the root-mean-square value of the original undistorted sentence and the final distorted sentences. This resulted into four channel conditions: 1 channel, 4 channels, 6 channels, and 8 channels. The 1-channel noise-vocoding provides a baseline condition as speech encoded with only one frequency band is least to non-intelligible. However, speech vocoded through four or more channels has been shown to be well intelligible – Ueda and Nakajima (2017) derived from the factor analysis of spectral fluctuations in eight languages, including German, that four channels are sufficient, and they also identified the optimal boundary frequencies for 4 channels vocoding. These are similar, although not identical, to the cochlear-frequency position function-based boundary frequencies chosen in the current study.

**Table 1:** Boundary frequencies (in Hz) for 1, 4, 6, and 8 channels noise-vocoding conditions.

Number of channels	Boundary frequencies								
1	70	9,000							
4	70	423	1,304	3,504	9,000				
6	70	268	633	1,304	2,539	4,813	9,000		
8	70	207	423	764	1,304	2,156	3,504	5,634	9,000

In the *unpredictable channel* context, each participant was presented with 120 unique sentences: 40 high-predictability, 40 medium-predictability, and 40 low-predictability sentences. Channel condition was also balanced across each sentence type; i.e., in each of high-, medium-, and low-predictability sentences, 10 sentences passed through each noise-vocoding channels – 1, 4, 6, and 8 – were presented. This resulted into 12 experimental lists. The sentences in each list were pseudo-randomized, that is, not more than three sentences of same channel condition, or same predictability condition appeared consecutively. This randomization ascertained uncertainty of next-trial speech quality/degradation in the global context of the experiment.

The same set of stimuli and experimental lists were used in the predictable channel context. Each participant was presented with 120 unique sentences blocked by channel conditions. There were four blocks of stimuli. Thirty sentences were presented in each of the four blocks. In the first block, all sentences were of 8 channels, followed by blocks of 6 channels, 4 channels, and 1 channel speech, consecutively (Sheldon et al., 2008). Within each block, 10 high-predictability, 10 medium-predictability, and 10 low-predictability sentences were presented. All the sentences were pseudo-randomized so that not more than three sentences of the same predictability condition appeared consecutively in each block.

### Procedure

Participants were asked to use headphones or earphones. A prompt to adjust loudness was displayed at the beginning of the experiment: A noise-vocoded sound not used in the main experiment was presented, and participants were asked to adjust the loudness at their level of comfort. One spoken sentence was presented in each trial. Eight practice trials were presented before presenting 120 experimental trials. They were asked to enter what they had heard (i.e., to type in the entire sentence) *via* keyboard. Guessing was encouraged. At the end of each trial, they were asked to judge their confidence in their response on a scale of 1 to 4, 1 being “just guessing” to 4 being “highly confident.” The response was not timed. The experiment was about 40min long.

### Analyses

In the sentences used in our experiment, verbs evoke predictability of the sentence-final noun. Therefore, the effect of predictability (evoked by the verb) on language comprehension can be rightfully measured if we consider only those trials in which participants identify the verbs correctly. Verb-correct trials were considered as the sentence in which participants correctly understood the context (independent of whether they correctly understood the final target noun). Morphological inflections and typos were considered as correct. We first filtered out those trials in which context was not identified correctly, i.e., trials with incorrect verbs.^1^ Therefore, we excluded 2,469 out of 5,760 trials in unpredictable channel context and 2,374 out of 6,000 trials in predictable channel context from the analyses. The condition with 1-channel noise-vocoding was dropped from the analyses as there were no correct responses in any of the trials in this condition. The number of trials excluded per condition in each group is shown in Table 2.

**Table 2:** Number of trials excluded per condition.

Channel context	Channel condition	Predictability			Total trials presented
		Low	Medium	High	
Predictable (blocked design)	4 channels	208	181	215	1,500
	6 channels	62	60	49	1,500
	8 channels	32	29	38	1,500
Unpredictable (randomized design)	4 channels	251	236	241	1,440
	6 channels	61	63	55	1,440
	8 channels	49	31	42	1,440

Data preprocessing and analyses were performed in R-Studio (Version 3.6.1; R Core Team, 2019). Accuracy was analyzed with Generalized Linear Mixed Models with lmerTest (Kuznetsova et al., 2017) and lme4 (Bates et al., 2015b) packages which operate on log-odds scale. Binary responses (correct responses coded as 1 and incorrect responses coded as 0) for all participants in both groups (predictable global noise context and unpredictable global noise context) were fit with a binomial mixed-effects model; i.e., response accuracy was a categorical-dependent variable in the model (Jaeger, 2006, 2008). Channel condition (categorical; 4 channels, 6 channels, and 8 channels), target word predictability (categorical; high, medium, and low predictability), global channel context (categorical; predictable channel context and unpredictable channel context), and the interaction of channel condition and target word predictability, and the main effect of global channel context were included in the fixed effects.

We first fitted a model with maximal random effects structure that included random intercepts for each participant and item (Barr et al., 2013). Both by-participant and by-item random slopes were included for channel condition, target word predictability, and their interaction. To find the optimal model for the data, non-significant higher-order interactions were excluded from the fixed-effects structure (and from the random-effects structure) in a stepwise manner. Model selection was based on Akaike Information Criterion (Grueber et al., 2011; Richards et al., 2011) unless otherwise stated. Random effects not supported by the data that explained zero variance according to singular value decomposition were excluded to prevent overparameterization. This gave a more parsimonious model (Bates et al., 2015a). Such a model was then extended separately with: (i) item-related correlation parameters, (ii) participant-related correlation parameter, and (iii) both item- and participant-related correlation parameters. The best fitting model among the parsimonious and extended models was then selected as the optimal model for our data. Note that the parsimonious model shows qualitatively the same effects as the maximal model.

We applied treatment contrast for channel condition (8 channels as the baseline; factor levels: 8 channels, 4 channels, and 6 channels) and sliding difference contrast for target word predictability (factor levels: medium predictability, low predictability, and high predictability) and global channel context (factor levels: unpredictable and predictable). We report the results from the optimal model (see Table 3).

**Table 3:** Estimated effects of the best fitting generalized (binomial logistic) mixed-effects model accounting for the correct word recognition.

Fixed effects	Estimate	Standard error	Value of *z*	Value of *p*
Intercept	5.09	0.24	21.38	<0.001
Channel condition (4 channels)	−2.87	0.22	−13.10	<0.001
Channel condition (6 channels)	−0.66	0.19	−3.42	0.001
Target word predictability (Low-Medium)	−0.52	0.27	−1.97	0.049
Target word predictability (High-Low)	2.18	0.30	7.21	<0.001
Channel condition×Target word predictability	−0.71	0.29	−2.44	0.015
Global channel context (Unpredictable - Predictable)	−0.27	0.14	−2.02	0.043

*Optimal model: glmer(response~1+4ch+6ch+Low-Medium+High-Low+4ch: Low-Medium+ChannelContext+(1+4ch+High-Low | subject)+(1+4ch+6ch+Low-Medium+High-Low+4ch: Low-Medium || item)*.

*NB: The minus sign is only a symbolic representation of the difference, between two factors, from the sliding difference contrasts; see*Supplementary Material*for the details about the model description*.

*“ch” is an abbreviation for “channels.”*

## Results

**Figure 2:**
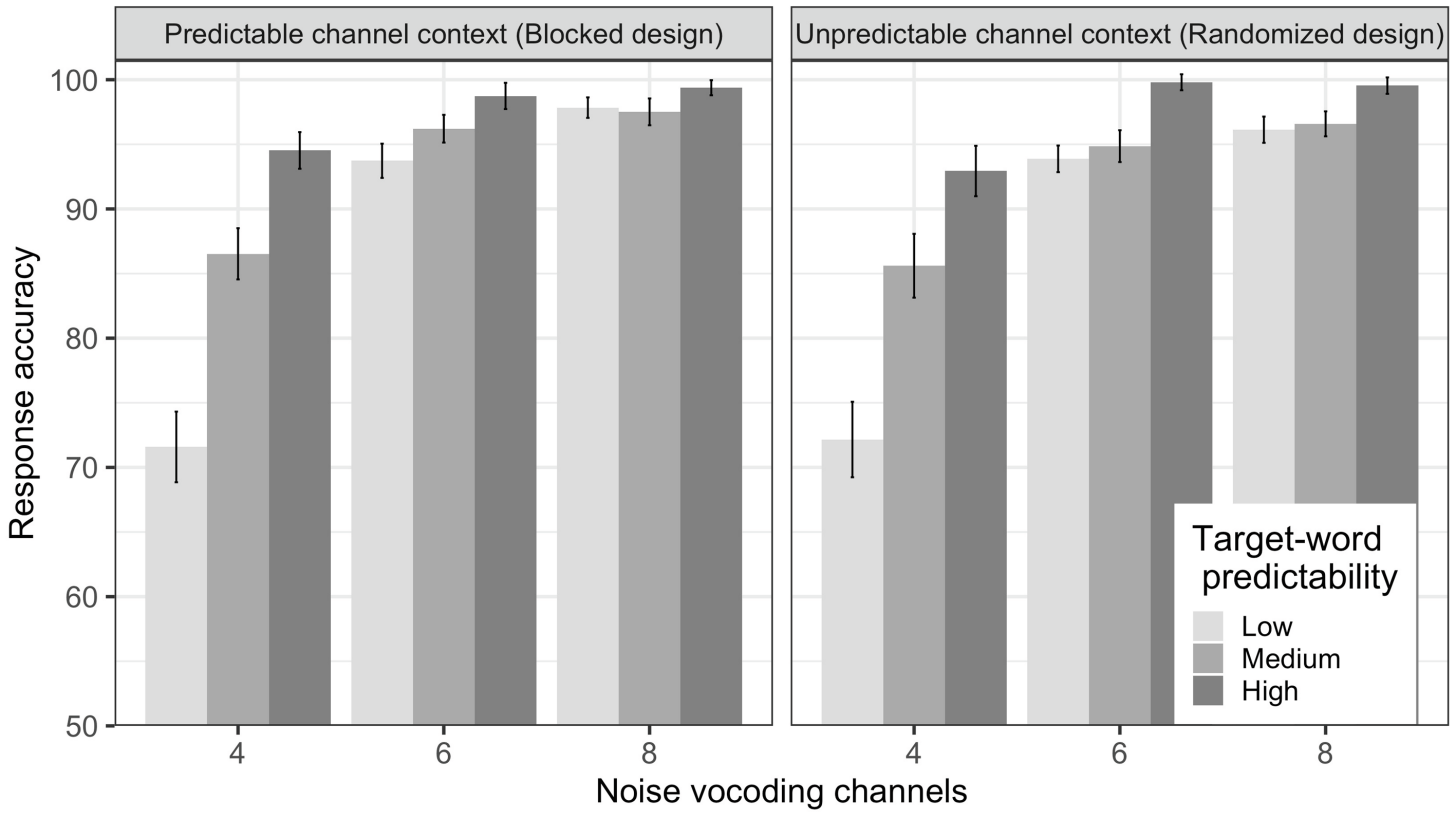
Mean response accuracy for the blocked design (left) and the randomized design (right). Error bars represent standard error of the means.

We primarily wanted to test (i) whether predictability facilitates language comprehension only at a moderate level of spectral degradation and (ii) whether adaptation to degraded speech influences language comprehension. We observed that the mean response accuracy increased with an increase in number of noise-vocoding channels from 4 to 6 to 8, and with an increase in target word predictability from low to medium to high, as can be seen in Figure 2. This trend is consistent across both predictable channel context (blocked design) and unpredictable channel context (randomized design; see also Tables 4 and 5).

**Table 4:** Mean response accuracy in predictable channel context.

Noise-vocoding	Predictability	Accuracy	SE
4 channels	Low	71.59	2.74
	Medium	86.53	1.99
	High	94.53	1.42
6 channels	Low	93.73	1.33
	Medium	96.21	1.08
	High	98.75	1.02
8 channels	Low	97.84	0.8
	Medium	97.52	1.04
	High	99.38	0.59

**Table 5:** Mean response accuracy in unpredictable channel context.

Noise-vocoding	Predictability	Accuracy	SE
4 channels	Low	72.16	2.93
	Medium	85.61	2.47
	High	92.94	1.96
6 channels	Low	93.88	1.04
	Medium	94.86	1.24
	High	99.81	0.62
8 channels	Low	96.14	1.02
	Medium	96.59	0.97
	High	99.55	0.64

The results of statistical analysis confirmed these observations. It showed that there was a main effect of channel condition indicating that the response accuracy was higher in the 8 channels than in the 4 channels [*β* =−2.87, SE=0.22, *z* (6917)=−13.10, *p* <0.001] and 6 channels [*β* =−0.66, SE=0.19, *z* (6917)=−3.42, *p* <0.001]. There was a main effect of target word predictability, suggesting that response accuracy was lower at low predictability than both high-predictability [*β* =2.18, SE=0.30, *z* (6917)=7.21, *p* <0.001] and medium-predictability sentences [*β* =−0.52, SE=0.27, *z* (6917)=−1.97, *p* =0.049]. There was also an interaction between channel condition and target word predictability [*β* =−0.71, SE=0.29, *z* (6917)=−2.44, *p* =0.015]. See Figure 2 for the corresponding mean accuracies.

Subsequent subgroup analyses were performed following the same procedure as described above. The results are shown in Table 6. They revealed that the interaction was driven by the effect of predictability at 4 channels: The accuracy at high-predictability sentences was higher than medium-predictability sentences [*β* =1.14, SE=0.37, *z* (1608)=3.10, *p* =0.002], which in turn was also higher than low-predictability sentences [*β* =1.01, SE=0.24, *z* (1608)=4.20, *p* <0.001]. There was no significant difference in response accuracy between low- and medium-predictability sentences at both 6 [*β* =0.33, SE=0.32, *z* (2590)=1.04, *p* =0.3] and 8 channels [*β* =−0.01, SE=0.32, *z* (2719)=−0.04, *p* =0.965]. However, response accuracy was higher in high-predictability than in medium-predictability sentences at both 6 channels [*β* =1.83, SE=0.65, *z* (2590)=2.83, *p* <0.005] and 8 channels [*β* =1.54, SE=0.61, *z* (2719)=2.54, *p* =0.011].

**Table 6:** Estimated effects of the generalized (binomial logistic) mixed-effects model accounting for the correct word recognition at 4 channels condition.

Fixed effects	Estimate	Standard error	Value of *z*	Value of *p*
Intercept	2.17	0.20	10.95	<0.001
Target word predictability (Medium-Low)	1.01	0.24	4.20	<0.001
Target word predictability (High-Medium)	1.14	0.37	3.10	0.002
Global channel context (Unpredictable - Predictable)	−0.27	0.18	−1.53	0.127

*Optimal model: glmer(response~1+4ch+6ch+Low-Medium+High-Low+4ch: Low-Medium+ChannelContext+(1+4ch+High-Low | subject)+(1+4ch+6ch+Low-Medium+High-Low+4ch: Low-Medium || item)*

*NB: The minus sign is only a symbolic representation of the difference, between two factors, from the sliding difference contrasts; see*Supplementary Material*for the details about the model description*.

*“ch” is an abbreviation for “channels.”*

We also found a main effect of global channel context showing that response accuracy was higher in predictable than in unpredictable channel context [*β* =−0.27, SE=0.14, *z* (6917)=−2.02, *p* =0.043].

To further test the effect of practice in the adaptation to noise, we added trial number as a fixed effect in the maximal model. Note that there were 30 trials in each block in the blocked design (predictable channel context). For comparability, we divided randomized design (unpredictable channel context) into four blocks; there were 30 consecutive trials in each block. Then, following the same procedure as above, an optimal model was obtained. The results showed that response accuracy did not change throughout the experiment [*β* =−0.0001, SE=0.01, *z* (6917)=−0.02, *p* =0.985]. It remained constant within each block in predictable channel context [*β* =−0.02, SE=0.01, *z* (3626)=−1.43, *p* =0.152] as well as in unpredictable channel context [*β* =0.01, SE=0.01, *z* (3291)=1.05, *p* =0.292].

## Discussion

The present study had three goals: (i) to examine whether previously reported facilitatory effect of semantic predictability is restricted to only highly predictable sentence endings; (ii) to assess the role of perceptual adaptation on the facilitation of language comprehension by sentence predictability; and (iii) to use and establish a sensitive metric to measure language comprehension that takes into account whether listeners benefited from the semantic context of the sentence they have listened to.

Results of our study showed the expected interaction between predictability and degraded speech; that is, language comprehension was better for high-cloze than for low-cloze target words when the speech signal was moderately degraded by noise-vocoding through 4 channels, while the effect of predictability was absent when speech was not intelligible (noise-vocoding through 1 channel). These results are fully in line with Obleser and Kotz (2010); we partly included identical sentences from their study in the present study (see Supplementary Material). Importantly, in contrast to their study, we had also created sentences with medium-cloze target words (which were intermediate between high-cloze and low-cloze target words) and found that the effect of predictability was also significant when comparing sentences with medium-cloze target words against sentences with low-cloze target words at 4 channels noise-vocoding condition. Recognition of a target word was dependent on its level of predictability (measured by cloze probability), and correct recognition was not just limited to high-cloze target words. These significant differences in response accuracy between medium-cloze and low-cloze target words, and between medium-cloze and high-cloze target words at noise-vocoding through 4 channels show that the sentence-final word recognition is facilitated by semantic predictability in a *graded manner*. This is in line with the findings from the ERP literature where it has been observed that semantic predictability, in terms of cloze probability of target word of a sentence, modulates semantic processing, indexed by N400, in a graded manner (DeLong et al., 2005; Wlotko and Federmeier, 2012; Nieuwland et al., 2018).

The interpretation of the observed graded effect of semantic predictability at the moderate level of spectral degradation (i.e., at noise-vocoding through 4 channels) provides a novel insight into how listeners form prediction when the bottom-up input is compromised. That is, in an adverse listening condition, listeners rely more on top-down semantic prediction than on bottom-up acoustic-phonetic cues. However, such a reliance on top-down prediction is not an all-or-none phenomenon; instead, listeners form a probabilistic prediction of the target word. The effect of target word predictability on comprehension is not sharply focused solely on high-cloze target words like a “searchlight.” But rather it is spread across a wide range, including low-cloze and medium-cloze target words. As the cloze probability of the target words decreases from high to low, the focus of the searchlight becomes less precise.

Obleser et al. (2007) and Strauß et al. (2013) reported an effect of predictability on language comprehension at noise-vocoding through 8 channels. On the other hand, we and Obleser and Kotz (2010) find a similar effect in 4 channels. This can be explained by the relative complexity of the stimuli used in this latter study. Obleser et al. (2007) took the sentences from G-SPIN (German version of Speech in Noise) test. Those sentences are longer and do not have uniform form and structure, while the sentences in Obleser and Kotz (2010) and the current study are shorter and have a uniform form and structure (Subject-Verb-Object). Owing to this fact, noise-vocoding through 4 channels was not intelligible enough for the G-SPIN test sentences, and the effect of predictability could be observed only at a higher number of vocoding channels (8 channels). This difference in number of channels (4 vs. 8) required for the effect of predictability to emerge is, therefore, due to the difference in stimuli complexity; the moderate-level spectral degradation could either be 8 channels or 4 channels depending on stimuli complexity. In the present study, moderate level of spectral degradation could be observed at noise-vocoding through 4 channels.

Previously reported facilitatory effect of semantic predictability comes from studies conducted in laboratory setups. The current experiment was conducted online. There is a possibility that different participants used different hearing devices. Such variability in hearing devices could not be controlled for in our experiment although the participants were restricted to using only desktop/laptop computers. However, we have no reason to believe that these variance sources are systematically correlated with our between-group manipulation (global channel context) and the effects are constant within subjects. Moreover, the main finding of this study, i.e., the graded effect of semantic predictability, is observed in both the groups.

We highlight the importance of considering participants’ context use in experiments that attempt to answer the questions pertaining to the use of top-down predictive cues. Unless it is established that participants correctly understood the context, the findings are likely to be confounded by the trials in which the predictability condition is not controlled as participants did not understand the context correctly, and the target word is not predictable based on the misunderstood context. In those cases, correctly recognizing the target word does not necessarily mean that listeners made use of the context and it was top-down prediction that facilitated the comprehension. Similarly, in cases where the target word was wrong, and the context was also not understood, it does not mean that participants did not form prediction based on what they understood. Also, instructions can affect how participants direct their attention during the task – they might shift attention strategically only to the target word, if this is all that is required for the task; hence, task instructions can also be a confounding factor (Sanders and Astheimer, 2008; Astheimer and Sanders, 2009; Li et al., 2014).

An alternative explanation of our findings could be that the listeners “guessed” the verb after first correctly identifying the noun in a sentence, which a reviewer pointed out. We therefore conducted an additional analysis where we compared forward predictability effects (from verb to noun) to the size of backward predictability effects (correct identification of the noun based on the final verb). If the observed effect is simply a cloze guessing effect, then we would expect that both forward and backward predictability effects are similar in size. If, on the other hand, understanding the verb really helps to shape predictions of the upcoming noun, and this helps intelligibility, then the forward prediction effect should be larger. The results of this complementary analysis (see the Supplementary Material) support the findings of the main analysis reported in the Results. In the backward predictability analysis, there was no graded effect of predictability, and the backward effect of “guessing” the verb *jongliert* after recognizing the noun *Baelle*, if present at all, was smaller than the forward effect of predicting the noun after recognizing the verb in the sentence *Sie jongliert die Baelle*. This corroborates our argument that listeners in fact made use of the verb-evoked context to form predictions about upcoming noun, and not the other way around, in a graded manner when the speech was moderately degraded.

The results of the analyses of trial number on the effect of channel context to capture trial-by-trial perceptual adaptation showed that the response accuracy did not increase over the course of experiment. This suggests that listeners’ performance remained constant over the course of experiment regardless of the predictability of next-trial spectral degradation. Perceptual adaptation occurs when the perceptual system of a listener retunes itself to the sensory properties of the auditory signal which can be facilitated by higher-level lexical information or feedback (Goldstone, 1998; Mattys et al., 2012). We speculate that the trial-by-trial variability in the spectral resolution of the speech signal in the unpredictable channel context prevented perceptual adaptation. Although there was certainty about the quality of speech signal within a block in the predictable channel context, we did not observe trial-by-trial perceptual adaptation in this condition either. This is contrary to previous studies showing that listeners adapt to degraded speech when the global context of speech quality is predictable (e.g., Davis et al., 2005; Erb et al., 2013). However, the crucial difference between those studies and our study is the manipulation of target word predictability. For example, Erb et al. (2013) presented sentences with only low-predictability target words from the G-SPIN test. We, on the other hand, parametrically varied target word predictability from low to medium and high. Note that we presented target words in a randomized order in both channel contexts. This alone introduces trial-by-trial uncertainty in the predictable channel context and possibly hinders trial-by-trial perceptual adaptation. As Goldstone (1998, p. 588) notes – “one way in which perception becomes adapted to tasks and environments is by increasing the attention paid to perceptual dimensions and features that are important, and/or by decreasing attention to irrelevant [perceptual] dimensions and features” (see also, Gold and Watanabe, 2010). In our study, listeners probably paid more attention to semantic properties of the sentences (i.e., contextual cues and target word predictability) than to the perceptual properties (i.e., spectral resolution or speech quality) as we had instructed. We speculate this might have resulted in the absence of trial-by-trial perceptual adaptation to degraded speech, even when next-trial channel condition was predictable. However, one noteworthy finding is the higher accuracy in the predictable channel context than in the unpredictable channel context. We interpret the differences in task performance in these two channel contexts in terms of general adaptation at a longer timescale. Adaptation to trial-by-trial variability of stimuli property reflects adaptation at a shorter timescale; at a longer timescale, however, listeners adapt to the experimental condition in which stimuli properties change slowly (Atienza et al., 2002; for neurobiological account, see Whitmire and Stanley, 2016; Zhang and Chacron, 2016; Weber et al., 2019). In both predictable and unpredictable channel contexts, adaptation in the short timescale was hindered by trial-by-trial variation of either one (target word predictability) or both properties (target word predictability and channel condition) of the speech stimuli. However, listeners adapted to the vocoded speech in the longer timescale when there was a certainty of channel condition (in predictable channel context) at the level of global channel context.

## Conclusion

In conclusion, this study provides novel insights into predictive language processing when bottom-up signal quality is compromised and uncertain: We show that while processing moderately degraded speech, listeners form top-down predictions across a wide range of semantic space that is not restricted within highly predictable sentence endings. In contrast to the narrowed expectation view, comprehension of words ranging from low- to high-cloze probability, including medium-cloze probability, is facilitated in a graded manner while listening to a moderately degraded speech. We also found better speech comprehension when individuals were likely to have adapted to the noise condition in the blocked design compared to the randomized design. We did not find learning effects at the trial-to-trial level of perceptual adaption – it may be that the adaptation was hampered by variation in higher-level semantic features (i.e., target word predictability). We also argue that for the examination of semantic predictability effects during language comprehension, the analyses of response accuracy should be based on the trials in which context evoking words are correctly identified in the first place to make sure that listeners make use of the contextual cues instead of analyzing general word recognition scores.

## Data Availability Statement

The raw data supporting the conclusions of this article will be made available by the authors, without undue reservation.

## Ethics Statement

The studies involving human participants were reviewed and approved by the Deutsche Gesellschaft für Sprachwissenschaft (DGfS) Ethics Committee. The patients/participants provided their written informed consent to participate in this study.

## Author Contributions

PB, VD, and JK were involved in planning and designing of the study. PB analyzed the data and wrote all parts of the manuscript. VD and JK made the suggestions to the framing, structuring, and presentation of findings as well as on the interpretation of findings. All authors contributed to the article and approved the submitted version.

## Funding

This study was funded by the Deutsche Forschungsgemeinschaft (DFG, German Research Foundation) – Project-ID 232722074 – SFB 1102.

## Conflict of Interest

The authors declare that the research was conducted in the absence of any commercial or financial relationships that could be construed as a potential conflict of interest.

## Publisher’s Note

All claims expressed in this article are solely those of the authors and do not necessarily represent those of their affiliated organizations, or those of the publisher, the editors and the reviewers. Any product that may be evaluated in this article, or claim that may be made by its manufacturer, is not guaranteed or endorsed by the publisher.

## Footnotes

^1^We also performed a complementary analysis with only noun-correct trials, which was suggested by a reviewer, to test whether there was a backward effect of guessing the verb after first recognizing the noun in a sentence. The results of the analysis are presented in the Supplementary Material.
