# Supplementary material for: Semantic Predictability Facilitates Comprehension of Degraded Speech in a Graded Manner
Source: Front Psychol. 2021 Sep 9;12:714485. doi: 10.3389/fpsyg.2021.714485 (PMC8459870; doi:10.3389/fpsyg.2021.714485)
Supplement: Supplementary file 2 [file Data_Sheet_2.PDF]

Process of obtaining optimal model mentioned in the "Analyses" section:

First, we created a model matrix with the R function '*model.matrix(~channels*predictability, data=dataset*'. Therefore, the model matrix contained the columns representing the terms for the main effect of speech degradation, main effect of predictability, and their interactions. These columns were then explicitly mentioned in the fixed effect and random effect structure of the model.

The maximal model (M0) included all the columns of the model matrix and was specified as the zero-correlation-parameter model. It did converge. To obtain a parsimonious model optimal for the data, we excluded the interaction terms that were not significant ($z < 2$, $p < .05$) one at a time. The AIC of each succeeding model was compared with the preceding model to test if the model fit increased in each step. The model fit did not decrease with the exclusion of the non-significant higher order interaction terms. The model (M1) achieved at the end of this step included only the significant main effect terms and significant higher order interactions; it fit the data better in comparison to the model (M0) that included non-significant interaction terms.

To further obtain a parsimony, principal component analysis (PCA) was performed on the model thus achieved (M1). PCA of M1 showed that all the components in the random slopes for Items explained non-zero variance, but 3 out of 6 components in the random slopes for Participants explain zero variance, although M1 had lesser parameters than M0. A less complex model (M2) was created excluding the components from the random effects structure which explained zero variance (Bates et al., 2015). PCA on this model (M2) confirmed that all remaining components explained non-zero variance.

All the procedure described above was performed on the zero-correlation-parameter model that assumed no correlation between by-subject intercept and slope as well as between by-item intercept and slope. Now we extended the model (M2) by including i) item-related correlation parameter (M3), ii) subject-related correlation parameter (M4), and iii) both item- and subject-related correlation parameter (M5), all separately. These extended models were compared with the preceding model (M2). The model extended with subject-related correlation parameter (M4) had the smallest AIC and therefore was the best fitting, optimal model for our data. The statistical inferences (e.g., significance of main effects of speech degradation, predictability, and their interactions) drawn from the maximal model and the optimal model remain the same.

We followed the same approach to obtain the optimal model for subgroup analysis at 4 channels condition and in the complementary analysis.

Results of Complementary Analysis

The complementary analyses showed that the main effects of target word predictability, channel condition and their interactions are significant (Table S1). However, subsequent subgroup analyses (Table S2) showed that when listeners identified nouns correctly at 4 channels condition, their response accuracy (of verb recognition) in high predictability sentences was not different than in medium predictability sentences ($\beta = 0.19$, SE = 0.29, z (1878) = 0.66, $p = .51$). There was only a significant difference in accuracy between low predictability and medium predictability sentences ($\beta = 0.56$, SE = 0.28, z (1878) = 2.01, $p = .04$). Compared to the model estimates of accuracy in verb-correct trials (estimates $\beta = 1.14$ and $\beta = 1.01$), the accuracy for noun-correct trials (estimates $\beta = 0.19$ and $\beta = 0.56$) are smaller.

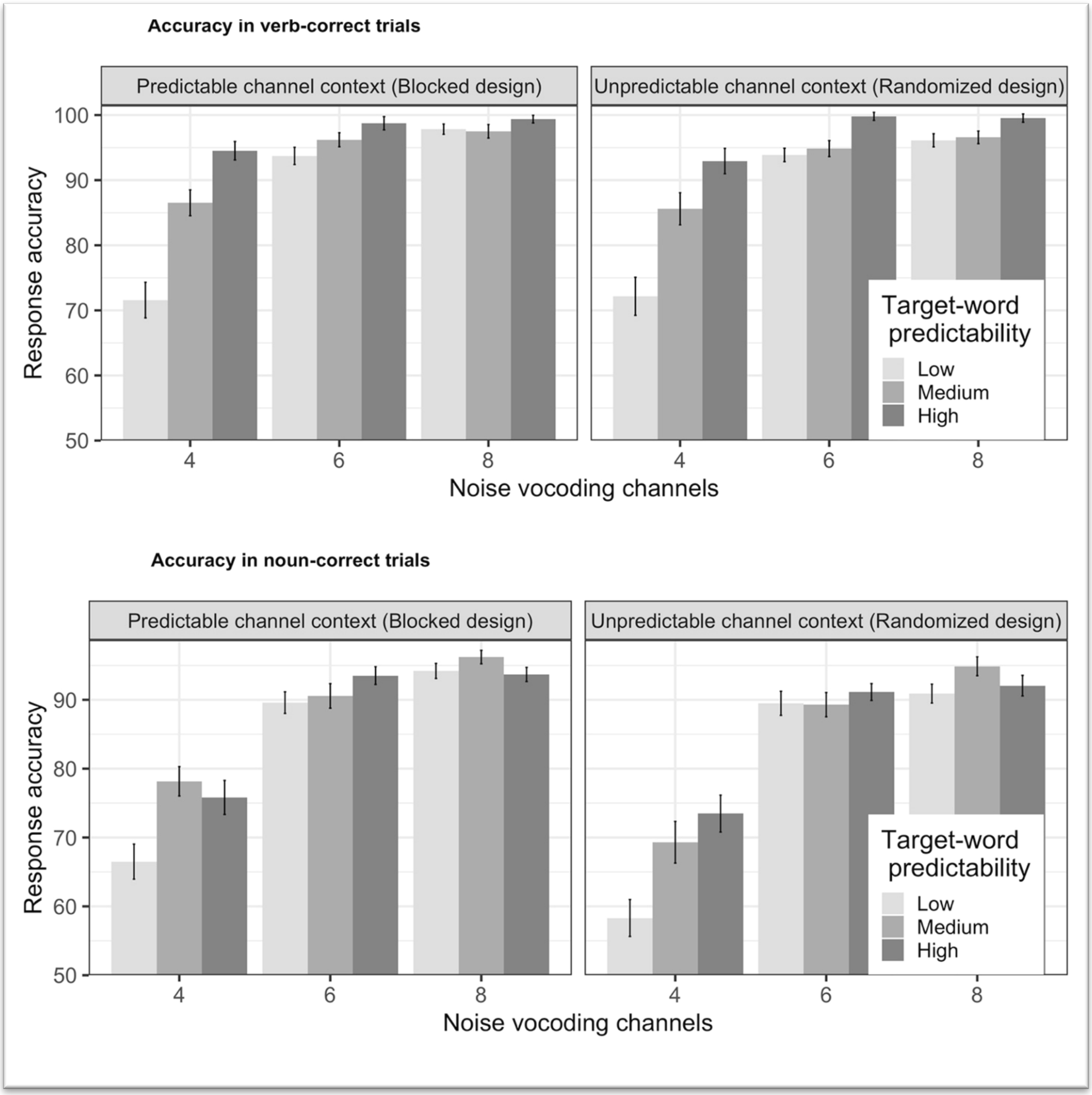

Figure S1. Top two panels show the response accuracy from the verb-correct trials (reported in the Analysis section), and the two panels in the bottom show response accuracy from the complementary analysis (noun-correct trials).

Table S1: Estimated effects of the best fitting generalized (binomial logistic) mixed effects model accounting for the correct verb-recognition in noun-correct trials

Fixed effects	Estimate	Standard error	*z* value	*p* value
Intercept	3.28	0.14	23.51	<.001
Channel condition (4 channels)	-2.13	0.13	-15.76	<.001
Channel condition (6 channels)	-0.35	0.14	-2.55	.011
Target word predictability (Low-Mid)	-0.72	0.28	-2.57	.01
Target word predictability (High-Low)	0.27	0.28	0.97	.33
Channel condition (4 channels) × Target word predictability (Low-Mid)	0.04	0.34	0.14	.89
Channel condition (6 channels) × Target word predictability (Low-Mid)	0.66	0.31	2.15	.03
Channel condition (4 channels) × Target word predictability (High-Low)	0.61	0.31	1.95	.05
Channel condition (6 channels) × Target word predictability (High-Low)	0.35	0.33	1.05	.29
Global channel context (Unpredictable - Predictable)	-0.37	0.13	-2.80	.005

Optimal model:
glmer(response ~ 1 + 4ch + 6ch + Low-Mid + High-Low + 4ch : Low-Mid + 6ch : Low-Mid + 4ch : High-Low + 6ch : High-Low + ChannelContext +
(1 + High-Low + 6ch : Low-Mid + 4ch : High-Low + 6ch : High-Low || subject) +
(1 + 4ch + 6ch + Low-Mid + High-Low + 4ch : Low-Mid + 6ch : Low-Mid + 4ch : High-Low + 6ch : High-Low || item)

NB: The minus sign is only a symbolic representation of the difference, between two factors,

from the sliding difference contrasts.
'ch' is an abbreviation for 'Channels'.

Table S2. Estimated effects of the generalized (binomial logistic) mixed effects model accounting for the correct verb recognition in noun-correct trials at 4 channels condition

Fixed effects	Estimate	Standard error	*z* value	*p* value
Intercept	1.17	0.13	8.90	<0.001
Target word predictability (Mid-Low)	0.56	0.28	2.01	.04
Target word predictability (High-Mid)	0.19	0.29	0.66	.51
Global channel context (Unpredictable - Predictable)	-0.42	0.14	-2.94	.003

Optimal model:
glmer(response ~ 1 + Mid-Low + High-Mid + ChannelContext +
(1 | subject) +
(1 + Mid-Low + High-Mid || item)

NB: The minus sign is only a symbolic representation of the difference, between two factors, from the sliding difference contrasts.
